# Post-traumatic Stress Disorder Symptoms and Its Predictors Among Healthcare Workers Following COVID-19 Pandemic in Southern Ethiopia: A Cross-Sectional Study

**DOI:** 10.3389/fpsyt.2021.818910

**Published:** 2022-01-04

**Authors:** Mohammed Ayalew, Bedilu Deribe, Yacob Abraham, Yared Reta, Fikru Tadesse, Semira Defar

**Affiliations:** ^1^School of Nursing, Hawassa University College of Medicine and Health Sciences, Hawassa, Ethiopia; ^2^Department of Midwifery, Hawassa University College of Medicine and Health Sciences, Hawassa, Ethiopia

**Keywords:** psychological trauma, PTSD symptoms, health care workers, COVID-19, Ethiopia

## Abstract

**Background:** COVID-19 causes immense psychological pressure on communities in addition to physical misery. There is currently a scarcity of data on the psychological impact of the COVID-19 epidemic on Ethiopian healthcare workers (HCWs). Therefore, this study was aimed to assess the post-traumatic stress disorder (PTSD) symptoms and its predictors following COVID-19 pandemic among healthcare workers (HCWs) in southern Ethiopia.

**Methods:** A hospital based cross-sectional study design was used among 387 randomly selected HCWs between September 25 and October 25, 2020 at four selected public hospitals in Sidama National Regional State, southern Ethiopia. Impact of Event Scale-Revised (IES-R) was used to collect data post-traumatic stress disorder (PTSD) symptoms. Logistic regression analyses with 95% CI were used to examine the relationship between independent and outcome variables.

**Result:** The prevalence of PTSD symptoms was found in 56.8% of participants. Significant factors that increase risk of PTSD symptoms were being female (AOR = 1.91, 95% CI = 1.19, 3.05), married (AOR = 1.87, 95% CI = 1.12, 3.14) and nurses (AOR = 3.31, 95% CI = 1.66, 6.63). On the other hand, HCWs working other than emergency unit such as inpatients/wards (AOR = 0.43, 95% CI = 0.24, 0.75), OPD (AOR = 0.48, 95% CI = 0.24, 0.97) and other units (AOR = 0.49, 95% CI = 0.25, 0.96) less likely to be affected by PTSD symptoms.

**Conclusion:** The current study showed high levels of PTSD symptoms as psychological challenges for HCWs. Sex, age, marital status, type of profession and working environment were significant factors for PTSD symptoms in HCWs during the pandemic. HCWs require mental health support during and after the pandemic.

## Introduction

The coronavirus disease (COVID-19) pandemic, which began in China, continues to pose a global health hazard (1). The COVID-19 outbreak was declared as a public health emergency of worldwide concern by the World Health Organization (WHO) on January 30, 2020 (2). Globally, there have been about 260 million confirmed cases and almost 5.2 million COVID-19 related deaths reported by the end of November, 2021 (3). In Ethiopia, there were about 371,000 confirmed COVID-19 cases and more than 6,700 deaths in the country by the end of November, 2021, with a 1.82% case fatality rate (4).

COVID-19's rapid spread around the world has placed significant strain on healthcare workers (HCWs) who are directly and indirectly combating the pandemic, potentially increasing the risk of negative mental health outcomes (5). COVID-19 causes significant psychological stress and other health-related issues in HCWs, as they are responsible for infected patients, have frequent interactions with patients' families/relatives, and are occasionally scrutinized by the public (6). HCWs also fear that they can develop COVID-19 themselves, because of the increased risk of exposure to the virus. They are concerned that the infection is brought home and passed on to families and friends (7). In addition, wearing protective equipment for extended periods of time causes breathing difficulties and limited access to toilets and water, resulting in physical and mental exhaustion (7). There are frequent reports of excessive job load, isolation and prejudice and so they are extremely prone to physical weariness, fear, emotional disturbance and sleep problems (8).

Previous studies indicated that health-related pandemic disasters have been linked to posttraumatic stress disorder (PTSD) symptoms (9–11). The COVID-19 pandemic has the potential to be a traumatic situation. According to the Diagnostic and Statistical Manual of Mental Disorders (DSM-5), the presence of symptoms from the following four symptom clusters is required for the diagnosis of PTSD: Intrusion symptoms associated with traumatic event(s); persistent avoidance of stimuli associated with traumatic event(s); negative changes in cognition and mood associated with traumatic event(s); and significant changes in arousal and reactivity associated with traumatic event(s) beginning or becoming more severe following the occurrence of traumatic event(s) (12).

The psychological pressure on HCWs dealing with COVID-19 is great (13) such as post-traumatic stress symptoms. According to a study done in China during the initial outbreak of COVID-19, 53.8% of respondents rated moderate to severe level of psychological distress (14). A study conducted in health care workers in Singapore reported that 7.7% for clinical concern of PTSD (15). The prevalence of symptoms of PTSD in Oslo, Norway were 28.9% among HCWs (16). About one-fourth (27.7%) had clinically important symptoms of post-traumatic stress among workers in Mexico in another study (17). A similar study in Chinese nurses showed that 16.8% had symptoms of PTSD (18). According to recent studies in Ethiopian HCWs, the prevalence of psychological distress or PTSD symptoms ranges from 51.6 to 78.3% (19–23).

HCWs who work in emergency rooms, intensive care units (ICU), and isolation wards are more likely to acquire psychological problems (24). According to a study conducted in Singapore, doctors and those who are single have a higher risk of developing psychiatric symptoms than nurses and those who are married, respectively (25). Moreover, lack of social support and communication, as well as maladaptive coping and a lack of training, are all major risk factors for developing psychological morbidities (24).

In a high-pressure, high-risk anti-pandemic situation, HCWs frequently experience a variety of psychological issues (26). As a result, psychological assessment and intervention in victims and rescuers, such as medical personnel and volunteers, are critical for pandemic control. This concept is beneficial not only for early actions and psychological intervention, but also for significantly improving pandemic control and accelerating social recovery (27). Therefore, the mental health of HCWs should be safeguarded, since this can impact the success of the healthcare delivery and the control of COVID-19 pandemic.

It is critical to have a reliable estimate of the prevalence of mental health problems among HCWs during the COVID-19 pandemic in order to prevent, identify, and treat it. Despite the fact that numerous studies on the psychological impact of HCWs during the pandemic have been conducted in various countries, there is still paucity of evidences in Ethiopia (19–23), particularly in the southern area. Thus, the aim of this study is to assess the PTSD symptoms and its predictors among healthcare workers (HCWs) during COVID-19 pandemic in southern Ethiopia.

Our findings could be useful in developing interventions to help HCWs cope with the COVID-19 pandemic and future outbreaks. Also, this could help government organizations and healthcare professionals protect the community's mental health as the COVID-19 pandemic spreads across Ethiopia.

## Materials and Methods

### Study Area

This study was conducted at selected public hospitals (Hawassa University Comprehensive Specialized Hospital (HUCSH), Adare General Hospital, Leku Primary Hospital and Yirgalem General Hospital) in Sidama National Regional State.

### Study Design and Period

Institution based cross sectional study design was conducted among HCWs between September 25 and October 25, 2020.

### Study Subject

This study was conducted among front line HCWs working in medical and surgical inpatient units, intensive care units, emergency departments, and outpatient units. In addition, non-frontline health professionals who are working at regular chronic care clinic, laboratory, pharmacy, delivery etc. units were included.

### Sample Size and Sampling Procedure

The required sample size was determined using single population proportion formula **n** **=** **(Z**_**α/2**_**)**^2*^ **p(1-p)/d**^**2**^, where n is the sample size, z is the standard normal score set at 1.96, d is the desired degree of accuracy and p is the estimated proportion of the target population. Due to the lack of previous research to inform our expected sample proportion (p), we use a value that gives our sample size maximum i.e., *p* = 0.5. Then by taking *P* = 50%. Z_α/2_ = 1.96 and w = 5%, the computed sample size was 384 and by taking 10% non-response rate, the total sample size computed was 422.

The overall sample size was proportionally allocated to each health institution. Then simple random sampling method was used to select the study participants by taking the lists from the human resource office of each respective health institution.

### Data Collection Methods

The English version of the Impact of Event Scale-Revised (IES-R) was used to collect data. The IES-R is a self-administered questionnaire that has been used to assess the psychological impact (PTSD symptoms) of a public health crisis within 1 week of exposure (28). This is a 22-item Likert scale questionnaire ranging from 0 (not at all) to 4 (extremely), with a total score between 0 and 88. It is composed of three subscales and aims to measure avoidance, intrusion, and hyperarousal symptoms (29). It had high levels of internal consistency (Intrusion: Cronbach's alpha = 0.87–0.94, Avoidance: Cronbach's alpha = 0.84–0.87, Hyperarousal: Cronbach's alpha = 0.79–0.91) and test-retest reliability was ranged from 0.89 to 0.94 (9, 30, 31). The total IES-R score was divided into normal (0–23), mild (9, 24–31), moderate (14, 31–33), and severe (>37) psychological impact (PTSD symptoms). A score of 24 or more considered as a cut-off score for the presence of PTSD symptoms or psychological trauma (31). The internal consistency or Cronbach's alpha of IES-R in this study was 0.94.

Four nurses were involved in data collection after receiving a 2-day intensive training on data collection techniques. A pre-test was performed in 5% of the sample to identify potential problems with data collection instruments and to ensure the consistency of the questionnaires. During the data collection process, supervisors checked each questionnaire for completeness on a daily basis.

### Data Processing and Analysis

Collected data were entered to Epi-data version 3.1 and exported to SPSS version 24 for windows for analysis. Descriptive statistics were used to identify distributions of socio-demographic characteristics of the study participants. The magnitude of psychological impact, were described as a percentage. Logistic regression analyses with 95% CI were used to see the association between each independent and outcome variable. Finally, those variables which showed statistical significance at *P* < 0.05 and 95% CI in the final model was reported as independently associated with psychological impact. The model fitness test was checked using the Hosmer and Lemeshow goodness of fit test.

## Results

### Socio-Demographic Characteristics

The study included 387 health professionals, with a 91.7% response rate. The remaining 35 questionnaires were incomplete and were not analyzed. The majority of study participants 227 (58.7%) were male, 233 (60.2%) were aged 26–35 years, nearly half 191 (49.4%) were married, about three-fourth 298 (77.0) had BSc degree, 230 (59.4%) had ≤ 5 years of experience, 224 (57.9%) were living with their family, half 197 (50.9%) were nurses by profession and about one-third 138 (35.7%) were working at emergency department. Socio-demographic characteristics were described in Table 1.

**Table 1:** Socio-demographic characteristics of the study participants during COVID-19 pandemic in selected hospitals of Sidama National Regional State, southern Ethiopia 2020 (*n* = 387).

**Variable**	**Category**	**Frequency**	**Percentage (%)**
Sex	Male	227	58.7
	Female	160	41.3
Age	<25 years	112	28.9
	26–35 years	233	60.2
	≥36 years	42	10.9
Marital status	Single	185	47.8
	Married	191	49.4
	Divorced	11	2.8
Religion	Protestant	189	48.8
	Orthodox	159	41.1
	Muslim	23	5.9
	Others*^a^*	16	4.1
Educational status	Diploma	47	12.1
	BSc degree/equivalent	298	77.0
	MSc degree or above	42	10.9
Average monthly income	<145 USD*	233	60.2
	>146 USD*	154	39.8
Work experience in years	≤ 5 years	230	59.4
	6–10 years	121	31.3
	≥11 years	36	9.3
Place of residence	Rural	72	18.6
	Urban	315	81.4
Living status	With family	224	57.9
	Alone	143	37.0
	With others	20	5.3
Profession	Physician	88	22.7
	Nurses	197	50.9
	Other professionals^b^	102	26.4
Working environment	Emergency	138	35.7
	Inpatient units	120	31.0
	OPD	58	15.0
	Others^c^	71	18.3

NB: *USD, United States Dollar;

a *Catholic, traditional*;

b *Public health officers, laboratory, midwives, pharmacists*;

c *Delivery, laboratory, pharmacy, ART clinics, TB clinics etc.; OPD-outpatient department; BSc- Bachelor of Science; MSc- Masters of Science*.

### Prevalence of PTSD Symptoms

**Figure 1:**
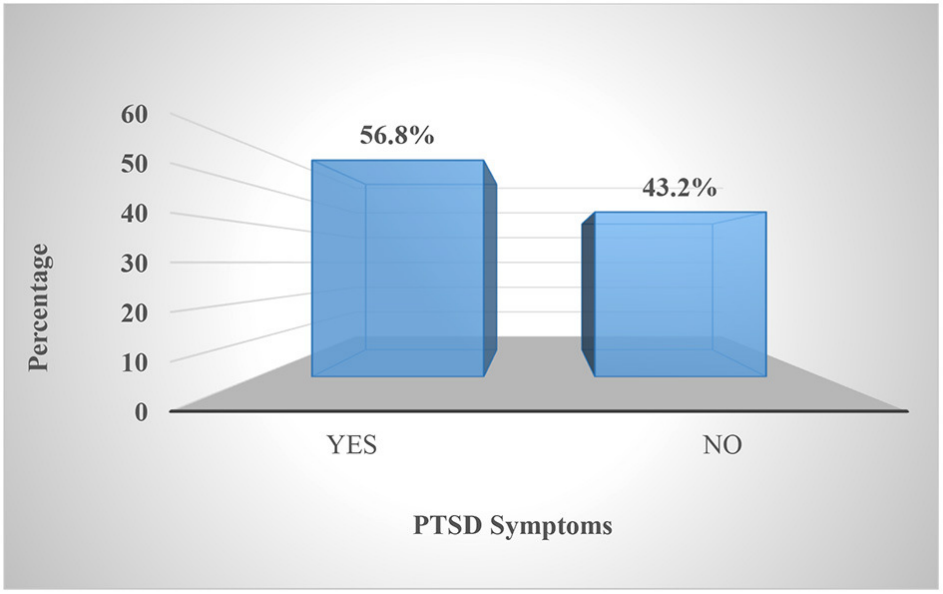
Prevalence of psychological impact (PTSD symptoms) of the study participants during COVID-19 pandemic in selected hospitals of Sidama National Regional State, southern Ethiopia, 2020 (*n* = 387).

The prevalence of PTSD symptoms was found to be 56.8% (Figure 1).

**Figure 2:**
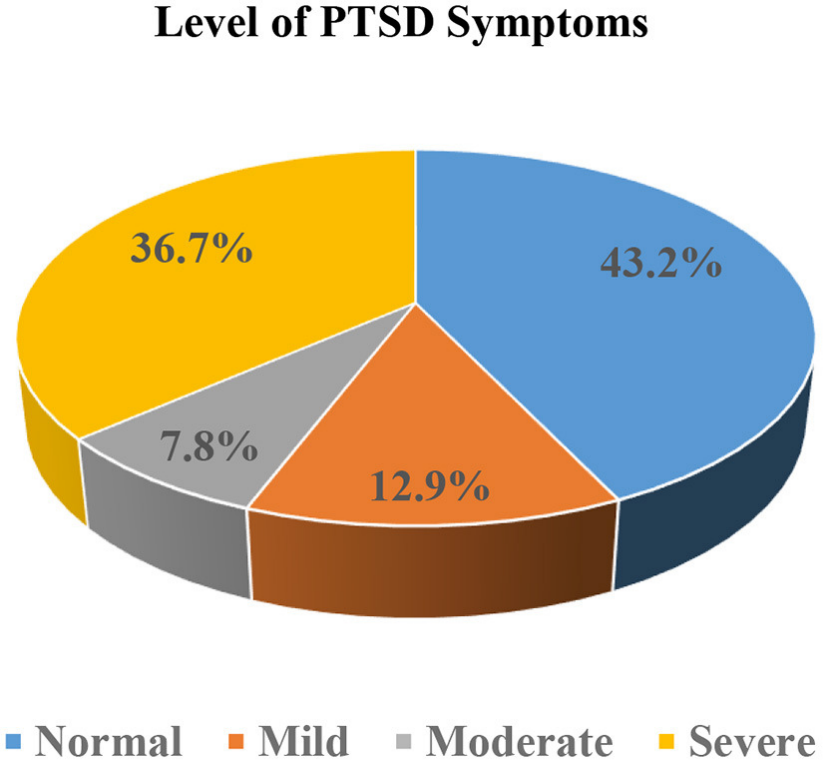
Severity level of PTSD symptoms of the study participants during COVID-19 pandemic in selected hospitals of Sidama National Regional State, southern Ethiopia, 2020 (*n* = 387).

Moreover, about one-third 142 (36.7%) of participants have severe, 28 (7.8%) have moderate and 50 (12.9%) mild level of PTSD symptoms as illustrated by Figure 2.

### Independent Predictors of PTSD Symptoms

Significant factors that increase risk of PTSD symptoms were being female (AOR = 1.91, 95% CI = 1.19, 3.05), married (AOR = 1.87, 95% CI = 1.12, 3.14) and nurses (AOR = 3.31, 95% CI = 1.66, 6.63). On the other hand, HCWs working other than emergency unit such as inpatients/wards (AOR = 0.43, 95% CI = 0.24, 0.75), OPD (AOR = 0.48, 95% CI = 0.24, 0.97) and other units (AOR = 0.49, 95% CI = 0.25, 0.96) less likely to be affected by PTSD symptoms (Table 2).

**Table 2:** Factors associated with PTSD symptoms of the study participants during COVID-19 pandemic in selected hospitals of Sidama National Regional State, southern Ethiopia, 2020 (*n* = 387).

**Variable**	**Category**	**PTSD symptoms**		**COR (95% CI)**	**AOR (95% CI)**
		**Yes**	**No**		
Age	<25 years	59	52	1	1
	26–35 years	142	103	1.09 (0.69, 1.72)	0.88 (0.50, 1.54)
	≥36 years	19	12	2.17 (1.01, 4.66)	1.36 (0.51, 3.67)
Sex	Male	111	116	1	1
	Female	109	51	2.23 (1.46, 3.41)	1.91 (1.19, 3.05)*
Marital status	Single	88	97	1	1
	Married	129	62	2.29 (1.51, 3.48)	1.87 (1.12, 3.14)*
	Divorced	3	8	0.41 (0.11, 1.61)	0.34 (0.07, 1.47)
Educational status	Diploma	34	13	2.38 (0.98, 5.73)	1.21 (0.43, 3.38)
	BSc degree	164	134	1.11 (0.58, 2.13)	0.68 (0.32, 1.44)
	MSc degree	22	20	1	1
Average monthly income	<145 USD	135	98	0.89 (0.59, 1.35)	0.89 (0.61, 1.57)
	>146 USD	85	69	1	1
Work experience in years	≤ 5 years	116	114	0.39 (0.18, 0.85)	0.81 (0.29, 2.20)
	6–10 years	78	43	0.69 (0.31, 1.58)	0.83 (0.32, 2.13)
	≥11 years	26	10	1	1
Living status	With family	137	87	1.28 (0.51, 3.24)	1.17 (0.69, 1.97)
	Alone	72	71	0.83 (0.32, 2.12)	1.40 (0.49, 4.01)
	With others	11	9	1	1
Profession	Physician	34	54	1	1
	Nurses	139	58	3.80 (2.25, 6.65)	3.31 (1.66, 6.63)*
	Other professionals*^a^*	47	55	1.36 (0.76, 2.42)	1.46 (0.74, 2.89)
Working environment	Emergency	96	42	1	1
	Inpatient units	60	60	0.52 (0.31, 0.88)	0.43 (0.24, 0.75)*
	OPD	28	30	0.50 (0.26, 0.94)	0.48 (0.24, 0.97)*
	Others^b^	36	35	0.62 (0.35, 1.11)	0.49 (0.25, 0.96)*

NB: *p-value < 0.05, USD, United States Dollar; OPD, outpatient department;

a *Public health officers, laboratory, midwives, pharmacists*;

b *Delivery, laboratory, pharmacy, ART clinics, TB clinics etc*.

## Discussion

Millions of people have died as a result of the COVID-19 pandemic, which has had a dramatic impact on the global population, and health-care providers have had to work a much busier schedule and for longer hours than predicted during this pandemic time. This study evaluates the severity of psychological trauma, known as post-traumatic stress disorder (PTSD), among health care workers during the COVID-19 pandemic in southern Ethiopia. It is added to a few other studies that have investigated this issue in Ethiopia (19–23).

The prevalence of PTSD symptoms was found to be 56.8% and more than one-third (36.7%) of participants had severe levels of PTSD symptoms. This finding is similar with recent studies in different parts of Ethiopia such as North Shoa (58%) (21), Northwest Ethiopia (55.1%) (19), and Gedeo Zone (51.6%) (22). In addition, studies from China (53.8%) (14), Italy (55%) (32), Spain (56.6%) (33), UK (60.6%) (34), and New York, US (57%) (35) reported similar findings to our study. But, a higher prevalence of PTSD (78%) were reported in southwest Ethiopia (20). Similarly a recent research by Zhang et al. found out that nearly three-fourth (73.4%) (IES-R ≥ 9) of study participants reported psychological trauma (36), which is higher than our finding. However, the prevalence of PTSD symptoms in this study was higher than in a study conducted in Oslo, Norway (28.9%) (16), Mexico (27.7%) (17), Italy (36.7%) (37), and Chinese nurses (16.8%) (18). Moreover, a recent literature review has shown that 11–74.4% of HCPs develop symptoms of PTSD (38). And also, several other investigations have shown very different results. Even if the same scale is employed, the use of diverse testing procedures and methodology in research, as well as the use of different classifications, contribute to widely disparate estimations of the prevalence of PTSD symptoms (39). In general, HCWs appear to be suffering from extensive mental health concerns during the COVID-19 epidemic (40, 41). Both before and after the epidemic, HCWs have a high demand for psychological care. The significant prevalence of psychological trauma confirmed with this study and other previous studies (19–23) suggests that HCWs in Ethiopia will require psychiatric care in the future.

Females were shown to have more likely experience PTSD symptoms in this study. This is backed up by a slew of studies showing that women are more likely than men to suffer from “internalizing” mental illnesses (42, 43). Male sex was independently related with a decreased prevalence of peritraumatic dissociative symptoms, according to a study by Azoulay et al. (44). Women were more likely than men to suffer from post-traumatic symptoms as a result of the stressful work environment (32). Furthermore, recent studies on COVID-19's health outcomes among HCWs reveal a high prevalence of mental health issues, particularly among women (45–47). On the one hand, women may have felt the pressure of working in the COVID-19 emergency more than their male counterparts because of the culturally bound double roles of women in the family, child-caring, and professional jobs. Women, on the other hand, place a higher value on their own internal experiences and the emotional states of others than men. Furthermore, a growing body of gender-specific research reveals that men use health-care services and report symptoms at a lower rate than women (48, 49).

In our study, we found out that the married HCWs were highly likely to experience PTSD symptoms than their counterparts. Similarly, PTSD symptoms were observed to be considerably greater among married employees in prior research conducted with healthcare professionals following COVID-19 outbreaks (50). Furthermore, in different studies conducted following the various outbreaks, married HCWs were found to be more concerned about their own health condition as well as the health of their families, leading to the conclusion that married HCWs experience more symptoms of psychological trauma (51, 52).

In comparison to physicians, nurses are more likely to experience PTSD symptoms, according to this study. Prior studies indicate that nurses are said to be at a higher risk of psychological disturbance or post-traumatic symptoms than doctors (19, 53–55). This may be due to nurses' workloads and night shifts, as well as being in contact with more risky patients than doctors (54). Of course, nursing personnel have longer and deeper interaction with COVID-19 patients, which provides the 24-h care, which raises the risk of psychological trauma, compared to other professionals (32). Physicians can be assumed to have certain unique somatization resistance which can be ascribed to their personal performance (56), professional experience, and self-awareness (57).

HCWs who worked in units other than emergency units were less likely to develop PTSD symptoms than those who worked in emergency units, according to our research. An Italian investigation came to a similar conclusion (32). Even though a patient's death is something that should be considered in any medical setting, especially in emergency rooms, it has been demonstrated to be one of the most common sources of stress for HCWs (58, 59). The emergency unit's so-called red zone, where most front-line HCWs perform their everyday operations, is a heavily contaminated and dangerous environment. Furthermore, doctors, nurses, and technicians may have lost a patient because to the pandemic or another medical issue. HCWs who saw the death of one of their COVID-19-infected patients, on the other hand, reported higher levels of psychological trauma (32). It is fact that, the death of a patient may remain an unspoken emotion, especially if overburdened by guilt and a sense of professional failure, which can influence the efficacy of physicians and other HCWs working with patients, resulting in significant adverse psychological consequences.

Even though, this study provided a baseline data, and we use a standardized tool IES-R designed to assess psychological trauma (PTSD symptoms), there are certain limitations to this research. To begin, PTSD symptoms were assessed solely by self-administered questionnaires rather than a psychiatric interview. Second, because we were unable to meet with HCWs face to face, we were unable to acquire extensive information about psycho-traumatic symptoms history. Third, this is a cross-sectional research paper. Fourth, no information on the type of mental health support provided to HCWs could be found. Therefore, longitudinal studies are needed to determine the prevalence of PTSD symptoms and its causative factors after the COVID-19 pandemic in the future researches.

## Conclusion

High levels of symptoms of PTSD symptoms emerged in the present study as psychological difficulties experienced by HCWs. Being female, married and nurses were significant factors associated with high risk of PTSD symptoms. Whereas, HCWs working other than emergency unit such as (inpatients, OPD and other units) were less likely to experience PTSD symptoms. Given the anticipated waves of COVID-19 and other healthcare crises, identifying risk factors for PTSD among HCWs and providing treatment for those who require it is crucial. Also, HCWs require psychiatric support at which monitoring and control can be performed during and after the pandemic. Furthermore, it is better if Ministry of Health and other concerned bodies provide mental health and psycho-social support (MHPSS) and arranging for in-service training to raise awareness for HCWs.

## Data Availability Statement

The original contributions presented in the study are included in the article/supplementary materials, further inquiries can be directed to the corresponding author/s.

## Ethics Statement

The studies involving human participants were reviewed and approved by Hawassa University, College of Medicine and Health Sciences, Institutional Review Board (IRB) with reference number IRB/295/12. The participants provided their written informed consent to participate in this study. The same permission letter was written by the university to each health institution. The purpose of the study was explained for the respondents and the right to withdraw from the study at any time was assured. Coding was used to eliminate names and other personal identification of respondents throughout the study process to ensure participants confidentiality. The patients/participants provided their written informed consent to participate in this study.

## Author Contributions

MA, BD, YA, YR, and FT participated in the conception and designed the study and involved in the data collection and analysis of the study. SD involved in the analysis of the study. MA prepares the manuscript for publication. BD, YA, YR, FT, and SD critically reviewed the manuscript. All authors read and approved the final manuscript.

## Conflict of Interest

The authors declare that the research was conducted in the absence of any commercial or financial relationships that could be construed as a potential conflict of interest.

## Publisher's Note

All claims expressed in this article are solely those of the authors and do not necessarily represent those of their affiliated organizations, or those of the publisher, the editors and the reviewers. Any product that may be evaluated in this article, or claim that may be made by its manufacturer, is not guaranteed or endorsed by the publisher.

## Abbreviations

AOR: Adjusted Odds Ratio
CI: Confidence Interval
COR: Crude Odds Ratio
COVID-19: the 2019 Coronavirus Disease
IES-R: Impact of Event Scale-Revised
HUCSH: Hawassa University Comprehensive Specialized Hospital
HCWs: Health Care Workers
OPD: Out Patient Department
PTSD: Post Traumatic Stress Disorder
WHO: World Health Organization.
